# Dietary Beta-Hydroxy-Beta-Methyl Butyrate Supplementation Affects Growth, Carcass Characteristics, Meat Quality, and Serum Metabolomics Profile in Broiler Chickens

**DOI:** 10.3389/fphys.2021.633964

**Published:** 2021-02-10

**Authors:** Zhiyi Tang, Bo Song, Changbing Zheng, Jie Zheng, Yulong Yin, Jiashun Chen

**Affiliations:** ^1^Animal Nutritional Genome and Germplasm Innovation Research Center, College of Animal Science and Technology, Hunan Agricultural University, Changsha, China; ^2^Key Laboratory of Agro-ecological Processes in Subtropical Region, Hunan Provincial Key Laboratory of Animal Nutritional Physiology and Metabolic Process, National Engineering Laboratory for Pollution Control and Waste Utilization in Livestock and Poultry Production, Institute of Subtropical Agriculture, Chinese Academy of Sciences, Changsha, China

**Keywords:** Wenshi chickens, beta-hydroxy-beta-methyl butyrate, growth performance, meat quality, serum metabolome

## Abstract

This study aimed to explore the effects of beta-hydroxy-beta-methyl butyrate (HMB) on serum metabolic profiles and meat quality of muscles in Wenshi broiler chickens. Birds were fed a basal diet with an additional 0, 0.05, 0.10, or 0.15% HMB, respectively. Results showed that dietary HMB quadratically increased the average daily gain (*P* = 0.058) and decreased feed:gain (*P* < 0.05) mainly in the starter phase. At 51 days of age, birds receiving 0.10% HMB diet exhibited less abdominal fat and more breast yield than the control (*P* < 0.05). Moreover, dietary HMB quadratically decreased the L^∗^ value and drip loss in selected muscles (*P* < 0.05) and increased the a^∗^ value in breast muscle (*P* < 0.05). Serum metabolome profiling showed that the most differentially abundant metabolites are lipids and lipid-like molecules, including phosphatidylcholines. It was concluded that HMB improved growth performance and meat quality of muscle in broilers.

## Introduction

As the fastest growing and most efficient meat species, broilers have been produced on a large scale to obtain more meat yield (Austic et al., 2013; Mir et al., 2017). Unfortunately, muscle abnormality and myopathy are subsequently induced by the fast growth rate of broilers, ultimately influencing meat quality (Mudalal et al., 2015; Tijare et al., 2016). Therefore, particular emphasis has been increasingly laid on improving meat quality of broiler meat in recent years (Cui et al., 2018; Cheng et al., 2019). In comparison with other edible meat (e.g., pork and beef), broiler meat is generally preferred by consumers owing to its relatively low cost and caloric value as well as high nutrient content (Brettonnet et al., 2010; Zhang et al., 2015). Since it is difficult to improve meat quality genetically in a short term, dietary manipulation is thus regarded as a relatively feasible practice.

β-Hydroxy-β-methylbutyrate (HMB), a metabolite of leucine, has gained more and more attention in the human and animal research field due to its regulatory roles in multibiological process and metabolism pathway. Specifically, in the field of poultry production, HMB has been reported to improve growth performance and carcass traits, promote muscle growth, enhance immune and intestinal function, and reduce mortality (Nissen et al., 1994; Qiao et al., 2013; Szczesniak et al., 2015; Suad et al., 2018). Despite these observations, little information is available regarding the effects of HMB on meat quality in broiler chicks. Therefore, further investigation is certainly warranted.

During the recent years, metabolomics approaches have been viewed as a powerful and valuable emerging tool for the assessment of meat quality (Yang et al., 2019; Wang et al., 2020). For example, liquid chromatography–mass spectrometry (LC-MS) was used to identify lipid-related metabolites in the breast muscle of Qingyuan chickens fed diets supplemented with mixed edible oils (Cui et al., 2020). ^1^H-NMR spectroscopy was performed to understand the relationship between metabolites and duck meat quality, suggesting the most relevant metabolites of duck breast meat quality (Wang et al., 2020). Therefore, this study aimed to explore the effects of diets supplemented with HMB on growth performance, carcass characteristics, and meat quality and further to identify the potential relationship between changes in serum metabolic profiles and meat quality traits of broiler chickens.

## Materials and Methods

### Ethics Statement

All experiments were carried out with the approval of the Animal Care and Use Committee of the Institute of Subtropical Agriculture, Chinese Academy of Sciences (ISA-2020-027). All birds were raised and maintained on a local commercial farm under the care of the Animal Care and Use Guidelines of Hunan Agricultural University.

### Animals and Diets

Three hundred thirty-six healthy 1-day-old male broiler chicks (Wenshi) with similar initial body weight (43.20 ± 0.56 g) were selected and randomly divided into four groups, in which there were 6 replicates and 14 birds per replicate. All birds were fed a basal diet (Table 1) supplemented with 0, 0.05, 0.10, or 0.15% HMB. HMB was provided by the TSI Jiyuan Group-Jiangyin Jiyuan Pharmaceutical Co., Ltd (Shanghai, China). Basal diets were formulated according to the nutrient requirements of the Chinese Feeding Standard of Chicken (Ministry of Agriculture of China, 2004). All birds were reared in the same well-ventilated poultry house with the same environmental conditions. The temperature was 33°C at days 1–7 and then decreased by 3°C/week until it reached 24°C. All birds had *ad libitum* access to diets and water throughout the 50-day experiment period.

**TABLE 1:** Composition and nutrient content of basal diets of broilers (air-dry basis, %).

Item	Starter phase Days 1–21	Grower phase Days 22–51
**Ingredients, %**		
Corn	56.49	63.45
Soybean meal	29.63	26.63
Corn gluten meal	0.70	0.32
Wheat bran	4.60	1.00
Soybean oil	4.40	4.00
L-Lysine HCl	0.26	0.26
DL-methionine	0.29	0.38
Threonine	0.09	0.10
Calcium hydrogen phosphate	1.70	2.00
Limestone	1.36	1.36
Premix^1^	0.48	0.50
**Nutrients (calculated value)**		
ME, MJ/kg	12.54	12.78
Crude protein	19.38	17.94
Calcium	1.00	1.05
Total phosphorus	0.66	0.68
Lysine	1.16	1.08
Methionine	0.59	0.66
Methionine + cystine	0.92	0.96

*^1^The premix provided per kilogram of diet: Vitamin A, 12,500 IU; Vitamin D_3_, 2500 IU; Vitamin E, 18.75 mg; Vitamin K_3_, 2.65 mg; Vitamin B_1_, 2 mg; Vitamin B_2_, 6 mg; Vitamin B_12_, 25 μg; pantothenic acid, 12 mg; nicotinic acid, 50 mg; biotin, 32.5 μg; folic acid, 1.25 mg; iron, 80 mg; zinc, 75 mg; manganese, 100 mg; iodine, 0.35 mg; copper, 8 mg; selenium, 0.15 mg.*

### Growth Performance and Carcass Characteristics

The birds were weighed and recorded on a pen basis during the starter phase (days 1–21) and during the grower phase (days 22–51). Feed intake was recorded at the end of each feeding phase (21 and 51 days). Based on these data, the average daily gain (ADG), average daily feed intake (ADFI), and the feed:gain (F:G) ratio were calculated for each feeding phase and for the overall rearing period.

At 51 days, two birds per replicate were randomly selected and killed after a 12 h fast to collect blood samples and to measure carcass weight as previously described (Wang et al., 2015). Upon removing the head, giblets, abdominal fat, and paws, eviscerated weight was recorded, and muscles of breast and leg as well as abdominal fat were weighed. Eviscerated yield percentages were calculated as the ratio between the eviscerated weight and the body weight after fasting. The weight percentage of abdominal fat were calculated as the percentage of body weight after fasting. Weight percentages of breast and leg muscles were calculated as percentages of eviscerated weight. Then, samples of breast and leg muscles were rapidly excised from the left side of the carcass and were then either stored at 4°C for meat quality measurement.

### Meat Quality Measurement

Meat quality was determined by measuring muscle color, pH, drip loss, cooking loss, and shear force of the breast and leg muscles as previously described (Zhang et al., 2015, 2018b).

### Serum Metabolomics Analysis

Serum samples (100 μl) were spiked with 20 μl of internal standards (0.3 mg/ml L-2-chlorophenylalanine in acetonitrile) and 400 μl of acetonitrile:methanol (1:1, v/v) solution and then extracted according to the manufacturer’s instructions (Majorbio Bio-Pharm Technology Co., Ltd., Shanghai, China). LC-MS/MS analysis for the extracted samples was performed using a Vanquish UHPLC system (Thermo Fisher) coupled with an Orbitrap Q Exactive HF-X mass spectrometer (Thermo Fisher Scientific, Inc) operating in the data-dependent acquisition mode. The quality control (QC) sample was a pooled sample in which aliquots of all the extracted samples were mixed and then analyzed using the same method as used for the analytic samples. It helped to represent the whole sample set, which would be injected at regular intervals (every eight samples) in order to monitor the stability of the analysis.

The parameters of chromatography were as follows: column, BEH C18 (100 mm × 2.1 mm i.d., 1.7 μm; Waters, Milford, United States); gradient mobile phase, water with 0.1% formic acid as solvent A and acetonitrile and isopropanol (1:1, v/v) (containing 0.1% formic acid) as solvent B; flowrate, 0.4 ml/min; sample injection volume, 2 μl; column temperature, 40°C. The gradient mobile phase program applied is as follows: *t* = 0 min, 95% A; *t* = 3 min, 80% A; *t* = 9 min, 5% A; *t* = 13.1 min, 95% A; *t* = 16 min, 95% A. The parameters of mass spectrum were as follows: sheath gas flowrate, 40 psi; aus gas flowrate, 10 psi; aus gas heater temperature, 400°C; ion-spray voltage floating (ISVF), -2800 V in negative mode and 3500 V in positive mode, respectively; and normalized collision energy, 20–40–60 V rolling for MS/MS. The detection was carried out over a mass range of 70–1050 m/z.

### Statistical Analysis

The data of growth performance, carcass characteristics, and meat quality were analyzed by one-way analysis of variance (ANOVA) using the SAS version 8.2 (SAS Institute Inc., Cary, NC, United States) software followed by Duncan’s multiple comparison test. Orthogonal polynomial contrasts were performed to determine linear and quadratic effects of increasing dietary HMB on the measured traits using the Statistical Package for Social Sciences (SPSS) version 18.0 software. Results are presented as means ± standard errors. Differences between significant means were considered statistically different at *P* < 0.05. Probability values between 0.05 and 0.10 were considered trends.

For the metabonomics data, the raw data were imported into the Progenesis QI software (Waters Corporation, Milford, United States) for data preprocessing after UHPLC-QE-HFX/MS analyses. In order to detect metabolites, we carried on the principal component analysis (PCA) and orthogonal partial least-squares (OPLS-DA) analysis. *T*-test (Student’s test) combined with the multivariate analysis of OPLS-DA was used to evaluate the differential metabolites or potential marker (variable importance in the projection (VIP) >1, *P* < 0.05), to reveal the differences in the metabolic composition and pathway among groups. Furthermore, the metabolite molecules were further identified by using the Human metabolome database (HMDB)^1^ and Metlin database^2^, and metabolism pathways were revealed by the iPath 3.0^3^ and the Kyoto Encyclopedia of Genes and Genomes (KEGG) pathway^4^. For analysis software, the online platform of Majorbio ISanger Cloud platform^5^ was used.

## Results

### Growth Performance and Carcass Qualities

As shown in Table 2, during the starter phase (days 1–21), chicks fed the 0.05, 0.10, or 0.15% HMB diet showed a comparably higher ADG and a lower F:G (*P* = 0.013) compared to chicks fed the control diet (*P* = 0.052), and the highest ADG and the lowest F:G were observed in the 0.10% group. The ADG increased and the F:G decreased quadratically in response to the increase in HMB supplementation (*P* = 0.058 and *P* = 0.002, respectively). No significant difference in ADFI was observed among the groups (*P* > 0.05). During the grower phase (days 22–51), 0.15% HMB led to a reduction in the ADG compared to the control group (*P* = 0.045). During the overall phase (days 1–51), ADG was the highest in the 0.10% HMB group and lowest in the 0.05 and 0.15% HMB groups, with an intermediate value in the control group (*P* < 0.05). ADFI tended to decrease linearly in response to the increase in HMB supplementation during the grower and overall phases (*P* = 0.076 and 0.067, respectively). No difference was observed in the F:G among the groups during the grower and overall phases (*P* > 0.05).

**TABLE 2:** Growth performance of broilers fed the diets with various levels of beta-hydroxy-beta-methyl butyrate (HMB).

Items	Dietary levels of HMB, %				SEM	*P*-values		
	0	0.05	0.10	0.15		ANOVA	Linear	Quadratic
BW at day 1, g	42.99	44.08	43.12	42.61	0.56	0.588	0.198	0.407
BW at day 51, g 1–21 days	1,000.03^*ab*^	954.27^*b*^	1,028.69^*a*^	935.72^*b*^	2.99	0.028	0.310	0.399
ADG, g/day	14.49^*b*^	14.94^*ab*^	15.63^*a*^	14.59^*b*^	0.35	0.052	0.520	0.058
ADFI, g/day	33.55	32.09	32.44	32.65	0.46	0.259	0.331	0.182
F:G 22–51 days	2.32^*a*^	2.15^*b*^	2.08^*b*^	2.24^*ab*^	0.14	0.013	0.212	0.002
ADG, g/day	22.24^*a*^	20.42^*ab*^	22.45^*a*^	19.90^*b*^	0.54	0.045	0.176	0.370
ADFI, g/day	112.26	106.38	102.28	100.16	1.44	0.370	0.076	0.200
F:G 1–51 days	5.09	5.25	4.59	5.03	0.34	0.430	0.526	0.735
ADG, g/day	19.14^*ab*^	18.23^*b*^	19.72^*a*^	17.87^*b*^	0.42	0.030	0.324	0.415
ADFI, g/day	72.90	69.24	67.36	66.40	1.03	0.323	0.067	0.168
F:G	3.82	3.81	3.43	3.71	0.25	0.284	0.336	0.421

*^*a,b*^Means with different superscripts within a row differ (*P* < 0.05).*

As shown in Table 3, no observable difference was noted in eviscerated yield and leg muscle yield upon HMB supplementation (*P* > 0.05). Breast muscle yield enhanced quadratically in response to dietary HMB supplementation (quadratic effect, *P* = 0.030), with the highest value observed in the 0.10% HMB group. The abdominal fat percentage was the highest in the 0.05% HMB group and lowest in the 0.10% HMB group, with intermediate values observed in the control and 0.15% HMB groups (*P* = 0.009).

**TABLE 3:** Carcass traits of broilers fed the diets with various levels of beta-hydroxy-beta-methyl butyrate (HMB).

Items	Dietary levels of HMB, %				SEM	*P-*values		
	0	0.05	0.10	0.15		ANOVA	Linear	Quadratic
Eviscerated yield, %	66.94	66.41	67.29	66.26	0.51	0.792	0.746	0.906
Breast muscle yield, %	17.38^*b*^	17.83^*b*^	18.86^*a*^	17.32^*b*^	0.33	0.014	0.630	0.030
Leg muscle yield, %	18.67	19.58	19.71	18.90	0.41	0.444	0.732	0.258
Abdominal fat, %	1.02^*bc*^	1.23^*a*^	0.93^*c*^	1.13^*ab*^	0.14	0.009	0.942	0.993

*^*a,b,c*^Means with different superscripts within a row differ (*P* < 0.05).*

### Meat Quality

The effects of dietary HMB on meat quality in breast and leg muscles are revealed in Table 4. In the breast muscle, the L^∗^ value, drip loss, cooking loss, and shear force decreased quadratically in response to dietary HMB supplementation (quadratic effect, *P* = 0.050, 0.023, 0.017, and 0.060, respectively), the minimum values of these parameters were obtained in the 0.10% HMB group. The values of a^∗^ and b^∗^ increased quadratically upon HMB supplementation (quadratic effect, *P* = 0.016 and 0.009, respectively), and the maximal value were observed in the 0.10 and 0.05% HMB groups, respectively. No significant differences were observed for the values of pH_45__*min*_ and pH_24__*h*_ among all groups (*P* > 0.05).

**TABLE 4:** Meat quality for the muscles of broilers fed the diets with various levels of beta-hydroxy-beta-methyl butyrate (HMB).

Items	Dietary levels of HMB, %				SEM	*P*-values		
	0	0.05	0.10	0.15		ANOVA	Linear	Quadratic
**Breast muscle**								
L* (lightness)	56.40^*a*^	55.67^*a*^	52.63^*b*^	54.96^*ab*^	0.54	0.019	0.078	0.050
a* (redness)	11.59^*a*^	11.74^*a*^	12.55^*a*^	10.12^*b*^	0.41	0.009	0.144	0.016
b* (yellowness)	10.03^*ab*^	10.92^*a*^	9.22^*bc*^	8.93^*c*^	0.35	0.002	0.007	0.009
pH_45__*min*_	5.79	5.80	5.92	5.79	0.17	0.675	0.715	0.672
pH_24__*h*_	5.64	5.60	5.65	5.58	0.18	0.185	0.258	0.458
Drip loss,%	6.55^*a*^	4.80^*b*^	4.95^*b*^	6.13^*ab*^	0.43	0.056	0.666	0.023
Cooking loss,%	24.13^*a*^	22.61^*a*^	19.98^*b*^	22.94^*a*^	0.54	0.011	0.149	0.017
Shear force, N	27.46^*a*^	19.47^*b*^	21.50^*b*^	22.34^*ab*^	0.77	0.075	0.195	0.060
**Leg muscle**								
L* (lightness)	54.42^*a*^	52.21^*b*^	52.00^*b*^	53.49^*ab*^	0.48	0.041	0.352	0.015
a* (redness)	15.67	15.58	15.75	15.73	0.37	0.990	0.832	0.974
b* (yellowness)	9.74^*a*^	8.01^*b*^	7.60^*b*^	8.04^*b*^	0.42	0.026	0.025	0.009
pH_45__*min*_	6.16^*b*^	6.17^*b*^	6.33^*a*^	6.24^*ab*^	0.13	0.057	0.077	0.129
pH_24__*h*_	6.05	6.04	6.16	6.10	0.12	0.192	0.173	0.346
Drip loss,%	5.61^*ab*^	3.74^*c*^	4.35^*bc*^	6.56^*a*^	0.41	0.001	0.199	<0.001
Cooking loss, %	28.03^*a*^	24.51^*a*^	19.99^*b*^	24.15^*a*^	0.67	0.002	0.021	0.002
Shear force, N	15.84	13.31	16.02	17.01	0.63	0.294	0.329	0.286

*^*a,b,c*^Means with different superscripts within a row differ (*P* < 0.05).*

In the leg muscle, the L^∗^ value, b^∗^ value, drip loss, and cooking loss decreased quadratically in response to dietary HMB supplementation (quadratic effect, *P* = 0.015, <0.001, and 0.002, respectively); the minimum values of these parameters except for drip loss were obtained in the 0.10% HMB group. The pH_45__*min*_ value tended to increase linearly in response to dietary HMB supplementation (quadratic effect, *P* = 0.077), and the maximum value was observed in the 0.10% HMB group. Dietary treatments did not significantly affect the values of a^∗^, pH_24__*h*_, and shear force (*P* > 0.05).

### Serum Metabolome

**FIGURE 1:**
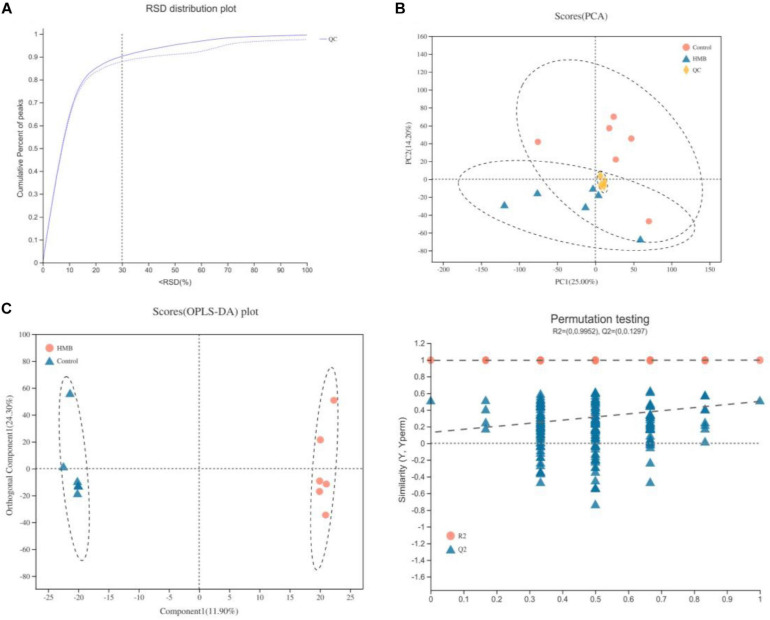
**(A)** Relative standard deviation (RSD) distribution of quality control samples for evaluating method repeatability. **(B)** Principal components analysis (PCA) of serum metabolites from chickens (*n* = 6) fed control diet or 0.10% beta-hydroxy-beta-methyl butyrate (HMB) diet. **(C)** Orthogonal correction partial least squares discriminant analysis (OPLS-DA) of serum metabolites from chickens (*n* = 6) fed control diet or 0.10% HMB diet.

A QC sample was injected every eight samples to verify the system stability during the whole sample batch. As shown in Figure 1A, >70% of the relative standard deviation (RSD) values of the QC samples were <30%, suggesting a good stability of the proposed method. Then, PCA and OPLS-DA were performed to further explore the slight differences in metabolic profiles among all groups. PCA data showed that the scatter plots of the serum were cross-distributed among the groups (Figure 1B). Unlike the PCA model, the OPLS-DA model displayed a clear separation between serum samples from birds fed control and HMB diets (Figure 1C), indicating a marked difference in serum metabolic patterns between the control and HMB groups. Furthermore, all the *Q*^2^ values were >0.4, indicating that the OPLS-DA was established successfully.

**FIGURE 2:**
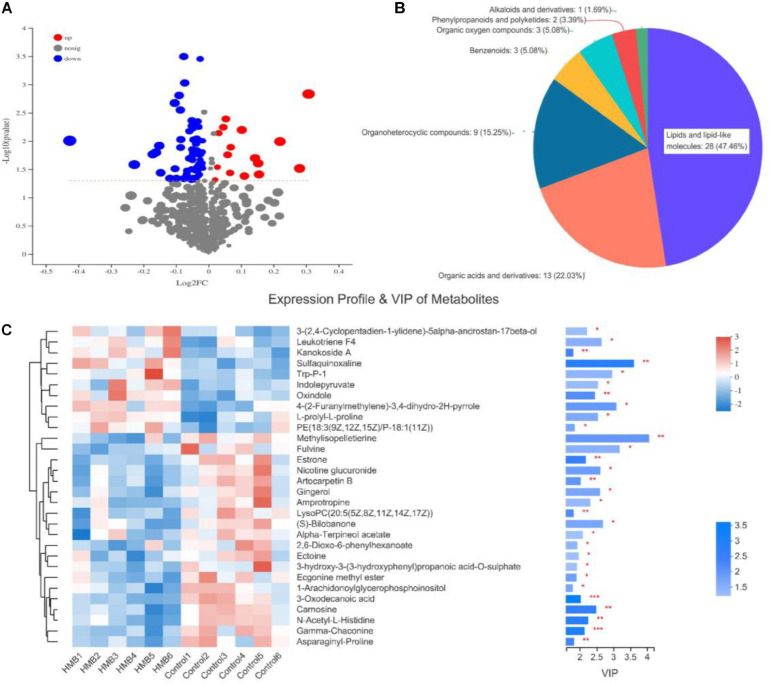
**(A)** Volcano plots of the identical biomarkers. Red and blue dots indicate upregulated and downregulated metabolites, respectively. Metabolites that showed no difference are shown in gray. **(B)** Relative abundance of serum metabolites of chickens fed control diet and 0.10% beta-hydroxy-beta-methyl butyrate (HMB) diet. **(C)** Hierarchical clustering analysis and heat maps of the 30 identified metabolites showing a significant difference between serum samples (*n* = 6) from chickens fed control diet and 0.10% HMB diet. Columns are discrete metabolites, and rows are individual samples, identified for the two diets. The color scale indicates the relative amounts of metabolites: red, higher levels; green, lower levels; white, unchanged. PE, phosphatidylethanolamine; LysoPC, lysophosphatidylcholines. Levels of significance are defined as *P < 0.05, **P < 0.01, and ***P < 0.001.

Of the 1941 metabolites shown to differ (VIP > 1 and *P* < 0.05) between the control and 0.10% HMB diets, 74 were annotated and identified based on the metabolome databases of HMDB (see text footnote 1) and Metlin (see text footnote 2). The blue compounds represent the downregulated biomarkers, and red represent the upregulated biomarkers (Figure 2A). The numbers of the HMDB compound metabolites are revealed in Figure 2B. Fifty-nine metabolites were present in high amounts and were divided in chemical categories as 28 lipids and lipid-like molecules, 13 organic acids and derivatives, 9 organoheterocyclic compounds, 3 benzenoids, 3 organic oxygen compounds, 2 phenylpropanoids and polyketides, and 1 alkaloids and derivatives. Then, to further investigate the differential metabolites with biological activities in each classification, a heat map was plotted for the control vs. 0.10% HMB chemical compositions (Figure 2C). Among the total, 10 compounds were elevated, and 20 compounds were reduced in the serum from chickens fed the 0.10% HMB rather than the control diet (VIP value > 1). The 10 elevated compounds were 3-(2,4-Cyclopentadien-1-ylidene)-5alpha-androstan-17beta-ol, leukotriene F4, kanokoside A, sulfaquinoxaline, Trp-P-1, indolepyruvate, oxindole, 4-(2-Furanylmethylene)-3,4-dihydro-2H-pyrrole, L-prolyl-L-proline, and PE[18:3(9Z,12Z,15Z)/ P-18:1(11Z)], which were predominantly eicosanoids, amino acids, peptides, and analogs, benzodiazines, indolyl carboxylic acids and derivatives, and glycerophosphoethanolamines.

**FIGURE 3:**
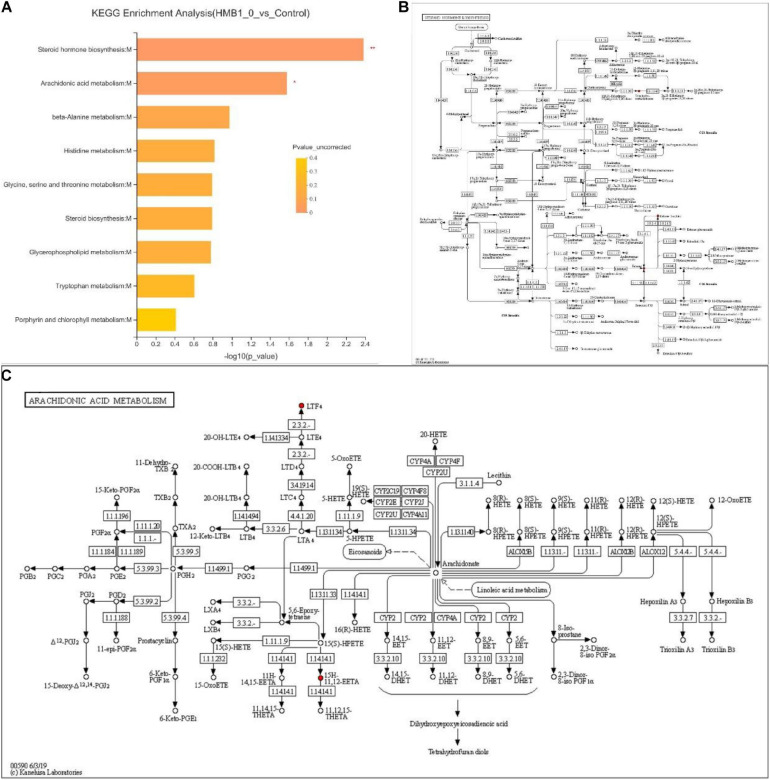
**(A)** Kyoto Encyclopedia of Genes and Genomes (KEGG) pathway enrichment data identified two significant pathways. **(B)** KEGG maps of steroid hormone biosynthesis:M. **(C)** KEGG maps of arachidonic acid metabolism:M. Levels of significance are defined as *P < 0.05 and **P < 0.01.

To determine the most evident and vital metabolic or biosynthetic pathways related to the metabolites, the metabolic pathways were identified according to the KEGG database. As shown in Figure 3, specific metabolites were mainly produced as a result of the steroid hormone biosynthesis:M and arachidonic acid metabolism:M (*P* < 0.01).

### The Relationship Between the Metabolites and Meat Quality

**FIGURE 4:**
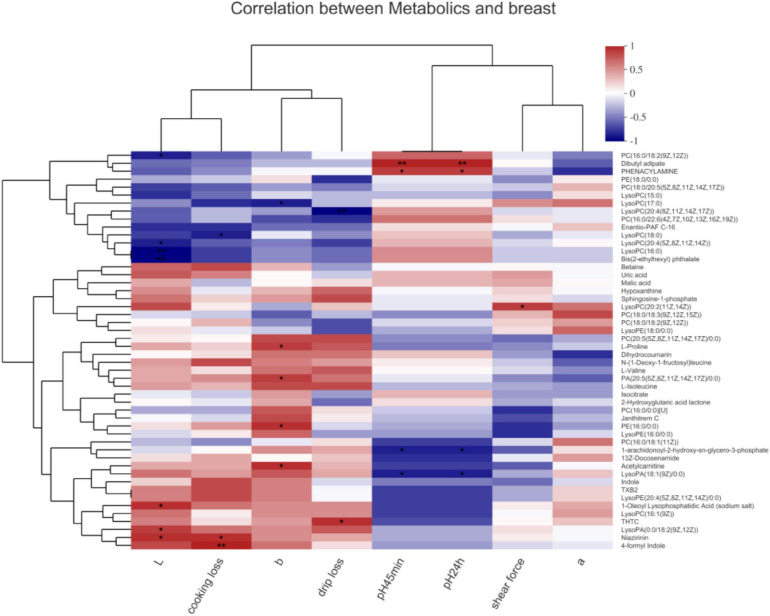
Correlations between metabolites and meat quality in the breast muscle of chickens. PC, phosphatidylcholine; PE, phosphatidylethanolamine; LysoPC, lysophosphatidylcholines. Levels of significance are defined as *P < 0.05 and **P < 0.01.

In the breast muscle (Figure 4), PC[16:0/18:2(9Z,12Z)], LysoPC[20:4(5Z,8Z,11Z,14Z)], LysoPC(16:0), and bis(2-ethylhexyl) phthalate negatively associated with the L^∗^ value (*P* < 0.05). 1-Oleoyl lysophosphatidic acid (sodium salt), lysoPA[0:0/18:2(9Z,12Z)], and niazirinin positively associated with the L^∗^ value (*P* < 0.05). LysoPC(17:0) negatively and L-proline, PA[20:5(5Z,8Z,11Z,14Z,17Z)/0:0)], PE(16:0/0:0), as well as acetylcarnitine positively associated with the b^∗^ value (*P* < 0.05). 2-n-Tetrahydrothiophenecarboxylic acid (THTC) positively associated with drip loss; niazirinin and 4-formyl indole positively associated with cooking loss (*P* < 0.05). LysoPC(18:0) negatively associated with cooking loss, and lysoPC(20:4(8Z,11Z,14Z,17Z) negatively associated with drip loss (*P* < 0.05). Dibutyl adipate and phenacylamine positively and 1-arachidonoyl-2-hydroxy-sn-glycero-3-phosphate together with lysoPA[18:1(9Z)/0:0] negatively correlated with the 45 min and 24 h values of pH (*P* < 0.05). LysoPC[20:2(11Z,14Z)] positively correlated with the shear force (*P* < 0.05).

**FIGURE 5:**
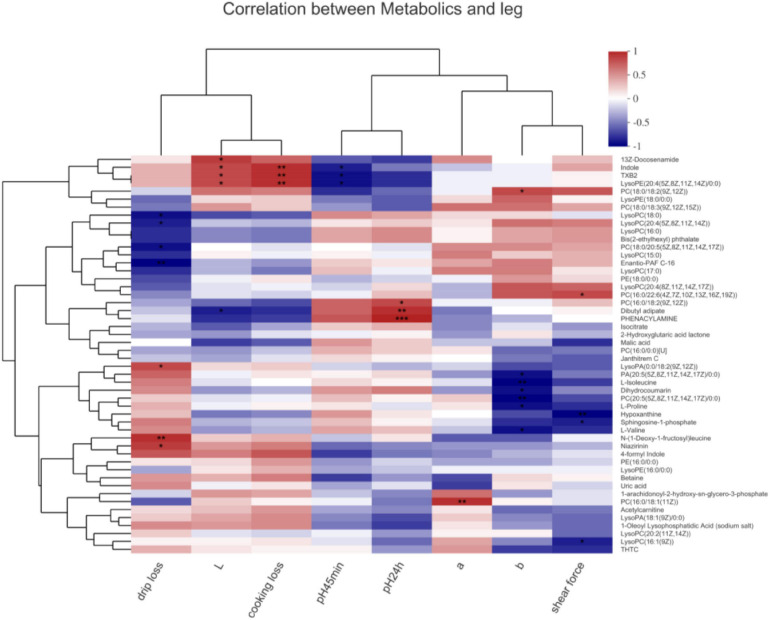
Correlations between metabolites and meat quality in the leg muscle of chickens. PA, phosphatidic acid; PC, phosphatidylcholine; PE, phosphatidylethanolamine; LysoPA, lysophosphatidic acid; LysoPC, lysophosphatidylcholines; LysoPE, lysophosphatidylethanolamine. Levels of significance are defined as *P < 0.05, **P < 0.01, and ***P < 0.001.

In the leg muscle (Figure 5), lysoPA[0:0/18:2(9Z,12Z)], N-(1-Deoxy-1-fructosyl)leucine, and niazirinin positively and lysoPC(18:0), PC[20:4(5Z,8Z,11Z,14Z)], PC[18:0/20:5(5Z, 8Z,11Z,14Z,17Z)], and enantio-PAF C-16 negatively associated with drip loss (*P* < 0.05). Indole, TXB2, and lysoPE[20:4(5Z,8Z,11Z,14Z/0:0)] positively associated with cooking loss (*P* < 0.01) and negatively correlated with pH_45__*min*_ (*P* < 0.05). 13Z-Docosenamide, indole, TXB2, and lysoPE[20:4(5Z,8Z,11Z,14Z/0:0)] positively associated with the L^∗^ value (*P* < 0.05). PC[16:0/18:2(9Z,12Z)], dibutyl adipate, and phenacylamine positively associated with pH_24__*h*_ (*P* < 0.05). PC[16:0/18:1(11Z)] positively associated with the a^∗^ value (*P* < 0.01). PC[18:0/18:2(9Z,12Z)] positively associated with the b^∗^ value (*P* < 0.05), dihydrocoumarin, PA[20:5(5Z,8Z,11Z,14Z,17Z)/0:0], isoleucine, PC[20:5(5Z,8Z, 11Z,14Z,17Z)/0:0], proline, and valine negatively associated with the b^∗^ value (*P* < 0.05). PC[16:0/22:6(4Z,7Z,10Z,13Z,16Z,19Z)] positively associated with the shear force (*P* < 0.05); hypoxanthine and sphingosine-1-phosphate negatively associated with the shear force (*P* < 0.05).

## Discussion

In the current study, 0.10% HMB supplementation markedly elevated ADG in the starter phase, without significant effects in the grower phase. Our current results fit well with previous studies reporting a similar elevation in ADG in response to 0.10% HMB supplementation in the starter phase (Qiao et al., 2013). Of note, increasing the level of dietary HMB to 0.15%, ADG was not affected in the starter phase but was significantly decreased in the grower phase. Moreover, dietary supplementation of HMB at the level of 0.05 and 0.10% improved F:G in the starter phase but not in the grower phase, and HMB was ineffective at the highest level (0.15%) in each feeding phase. A similar result was reported by previous studies in which muscle protein synthesis in neonatal pigs is maximally elevated at circulating HMB levels ∼90 nmol/ml, with higher concentrations of HMB not having an additional effect (Wheatley et al., 2014). Therefore, it appeared that concerning the growth performance, broilers in the starter phase were more sensitive to HMB, and the maximal effects were observed at the level of 0.10%, with negative effects in the grower phase at higher levels of HMB.

Similar to growth performance, the carcass traits of broilers were also significantly impacted by dietary HMB supplementation. In particular, HMB supplementation quadratically increased breast muscle yield, with the maximal effects obtained at the level of 0.10%, and exerted no effects on leg muscle yield. In agreement with this, previous studies have also shown that chicks fed 0.10% HMB diets exhibited more breast muscle yield at the end of each feeding phase (21 and 42 days of age), and no difference was observed in the leg muscle yield upon HMB supplementation (Qiao et al., 2013). These results suggested that, in comparison with the leg muscle, the breast muscle was more sensitive to HMB supplementation. The differences between the effects of the yields of breast muscle and leg muscle exerted by HMB might be related to the huge differences in the composition of muscle fiber types between breast muscle (mainly fast glycolytic type) and leg muscle (mainly slow oxidative type) (Zhang et al., 2020). Protein breakdown in muscles mainly containing fast glycolytic types was more inhibited by HMB than that in muscles mainly containing slow oxidative types (Ostaszewski et al., 2000).

Next, meat color, pH, water-holding capacity, and shear force were analyzed in this study to evaluate meat quality. The meat color is an indicator of meat freshness and quality and is generally assessed using L^∗^ (the measure of lightness), a^∗^ (the measure of redness), and b^∗^ (the measure of yellowness) (Liu et al., 2018). Water-holding capacity can be reflected by drip loss and cooking loss (Wan et al., 2017). Either a high drip loss or a high cooking loss indicates a decrease in water-holding capacity, which has the ability to induce liquid outflow, and loss of soluble nutrients and flavor (Ozturk et al., 2012). Moreover, reduced water-holding capacity can result in enhanced L^∗^ value (Zhang et al., 2018b). A higher L^∗^ value and a lower a^∗^ value suggest an increased occurrence of pale, soft, and exudative meat (Zhang et al., 2018a). Our current study showed that dietary HMB supplementation improved meat quality in both breast and leg muscles, as illustrated by decreased L^∗^ value and increased water-holding capacity as well as a^∗^ value, and the optimal level of HMB was at 0.10%.

Considering the importance of HMB in the nutritional value and quality of chicken meat, this study further made efforts to explore the metabolites and pathways behind the HMB diet altering the meat quality of chickens. The analyses of meat quality and serum metabolomics as affected by dietary HMB supplementation are rarely described in previous studies (Qiao et al., 2013; Ma et al., 2020). However, to better understand the interactions between diets and nutrition, more and more non-targeted metabolomics studies have been performed to assess the effects of other functional diets on metabolite content and pathways in the plasma or tissues (such as liver, muscle, and abdominal fat) of chicks (Huber et al., 2010; Ji et al., 2012; Baira et al., 2018; Cui et al., 2020). In this study, given the improvements of growth performance, carcass traits, and meat quality obtained in chickens fed the 0.10% HMB diet, serum samples from the 0.10% HMB and control diets were subjected to metabolomics analyses to find out the important differential metabolites and the enriched pathways. The stability and reliability of the metabolomics profiles obtained were supported by the results of RSD and OPLS-DA model.

A functional analysis of the differentially abundant metabolites was performed using HMDB. In the seven selected classifications, there were more predominantly accumulated lipids and lipid-like molecules, organic acids and derivatives, and organoheterocyclic compounds and less predominantly accumulated benzenoids, organic oxygen compounds, phenylpropanoids and polyketides, and alkaloids and derivatives. These results are in agreement with other authors who have identified lipids and lipid-like molecules, organic acids, and organoheterocyclic compounds as significant metabolites of microorganism growth (Chen et al., 2020; Cui et al., 2020). Next, a heat map was plotted for the control vs. 0.10% HMB diet to show the key differential metabolites with biological activities in each classification. Our results indicated that there was statistical difference mainly in the abundance of lipids and lipid-like molecules, including leukotriene F4, PE[18:3(9Z,12Z,15Z)/P-18:1(11Z)], estrone, lysoPC[20:5(5Z,8Z,11Z,14Z,17Z)], alpha-Terpineol acetate, 1-arachidonoylglycerophosphoinositol, and gamma-chaconine. In particular, leukotriene F4 and PE[18:3(9Z,12Z,15Z)/P-18:1(11Z)] were increased, and the remaining five metabolites were decreased in response to HMB supplementation. A further enrichment analysis indicated that diets significantly affected the pathways of steroid hormone biosynthesis:M and arachidonic acid metabolism:M. It has been reported that steroid hormones function as a growth-promoting agent in animals and also greatly affect the meat quality via altering the percentages of fat and protein in beef cattle (Fritsche and Steinhart, 1998). Arachidonic acid is associated with a diversity of biological effects including lipid homeostasis and muscle growth (Markworth and Cameron-Smith, 2013; Gol et al., 2018). It can also play key roles in maintaining membrane fluidity and in cell signaling when it is esterified into phospholipids (Wood et al., 2008). In addition, the results of OPLS-DA-based correlation analysis in the current study indicated that, in the breast muscle, PC metabolites negatively associated with the L^∗^ value, b^∗^ value, drip loss, and cooking loss; in the leg muscle, isoleucine, valine, dihydrocoumarin, and proline negatively associated with the b^∗^ value, and PC metabolites negatively associated with the drip loss. These PC metabolites were enriched and identified in the glycerophospholipid metabolism, which has gained much attention due to its profound influence on lipid metabolism and meat quality (Zhang et al., 2018c; Cui et al., 2020). Isoleucine and valine also have been the focus of considerable recent attention as supplements to improve meat quality of pigs (Xu et al., 2020). Although this study has established the mathematical correlations between these metabolites and meat quality, further work is needed to evaluate the biological relationships between them.

## Conclusion

In conclusion, our study concludes that dietary HMB supplementation improved the growth performance, carcass characteristics, and meat quality and regulated the related serum metabolites particularly through the pathways of steroid hormone biosynthesis:M and arachidonic acid metabolism:M in the broiler chicks, and the optimal level of HMB was 0.10%. In particular, the PC metabolites were strongly associated with the quality traits of breast meat, and isoleucine and valine were greatly related to those of leg meat. Therefore, these results may contribute to enhancing the understanding of metabolic mechanisms by which dietary HMB supplementation modulates meat quality of chickens. HMB is a potentially effective feed additive to obtain chicken meat with improved functional properties for consumers.

## Data Availability Statement

The original contributions presented in the study are included in the article/supplementary material, further inquiries can be directed to the corresponding author/s.

## Ethics Statement

All experiments were carried out with the approval of the Animal Care and Use Committee of the Institute of Subtropical Agriculture, Chinese Academy of Sciences (ISA-2020-027). All birds were raised and maintained on a local commercial farm under the care of the Animal Care and Use Guidelines of Hunan Agricultural University. Written informed consent was obtained from the owners for the participation of their animals in this study.

## Author Contributions

ZT and BS conducted most of the experiments, analyzed and interpreted the data, and wrote the manuscript. CZ and JZ conducted the research, analyzed and interpreted the data, and wrote the manuscript. YY and JC oversaw the study and reviewed the manuscript. JC supervised the study and reviewed the manuscript. All authors read and approved the final manuscript.

## Conflict of Interest

The authors declare that the research was conducted in the absence of any commercial or financial relationships that could be construed as a potential conflict of interest.

## Footnotes

1 http://www.hmdb.ca/

2 https://metlin.scripps.edu/

3 http://pathways.embl.de

4 http://www.genome.jp/kegg/

5 https://cloud.majorbio.com/
